# 3D printed cardiovascular models for surgical planning in complex congenital heart diseases

**DOI:** 10.1186/1532-429X-17-S1-P196

**Published:** 2015-02-03

**Authors:** Israel Valverde, Gorka Gomez, Cristina Suarez-Mejias, Amir-Reza Hosseinpour, Mark Hazekamp, Arno Roest, Jaime F Vazquez-Jimenez, Issam El-Rassi, Sergio Uribe, Tomas Gomez-Cia

**Affiliations:** Cardiovascular Pathology Unit, Institute of Biomedicine of Seville (IBIS), Seville, Spain; Paediatric Cardiology, Hospital Virgen del Rocio, Seville, Spain; Technological Innovation Group, Hospital Virgen del Rocio, Seville, Spain; Cardiac Surgery Unit, Hospital Virgen del Rocio, Seville, Spain; Department of Cardiothoracic Surgery, University Hospital Leiden, Leiden, Netherlands; Paediatric Cardiology, University Hospital Leiden, Leiden, Netherlands; Pediatric Cardiac Surgery, American University Hospital Medical Center, Beirut, Lebanon; Pediatric Cardiac Surgery, University Hospital RWTH Aachen, Aachen, Germany; Department of Radiology and Biomedical Imaging Center, Pontificia Universidad Católica de Chile, Santiago, Chile

## Background

A precise understanding of the anatomical structures of the heart and great vessels is essential for surgical planning in order to avoid unexpected findings. Rapid prototyping techniques are used to print three-dimensional (3D) replicas of patients' cardiovascular anatomy based on 3D clinical images such as MRI. The purpose of this study is to explore the use of 3D patient-specific cardiovascular models using rapid prototyping techniques to improve surgical planning in patients with complex congenital heart disease.

## Methods

**Figure 1:**
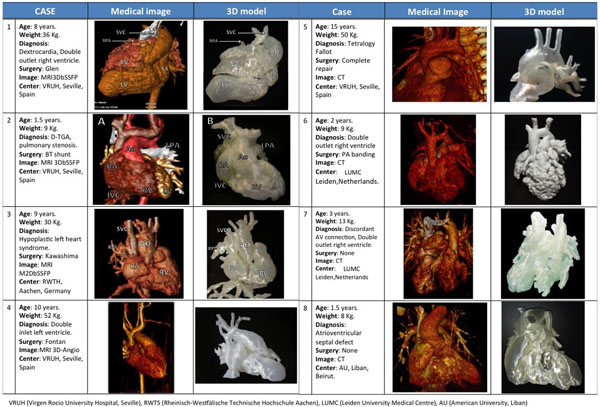
Study population demographics, medical images and 3D-models.

This European prospective multicenter study included 8 patients with complex congenital heart diseases (Figure 1). Magnetic resonance imaging (MRI) and computed tomography (CT) were used to acquire 3D cardiovascular anatomy. Images were segmented and 3D mesh was created using AYRA software (IKIRIA, Spain). Fused deposition technique using polylactic acid was used. A Bland-Altman analysis was used to evaluate the diameters measurement agreement between the 3D printed model and the patient's MRI and CT. 3D-models were used to plan the surgery. After the procedure, surgeons involved filled a questionnaire form to evaluate the usefulness of the 3D printed models to plan the surgery.

## Results

**Figure 2:**
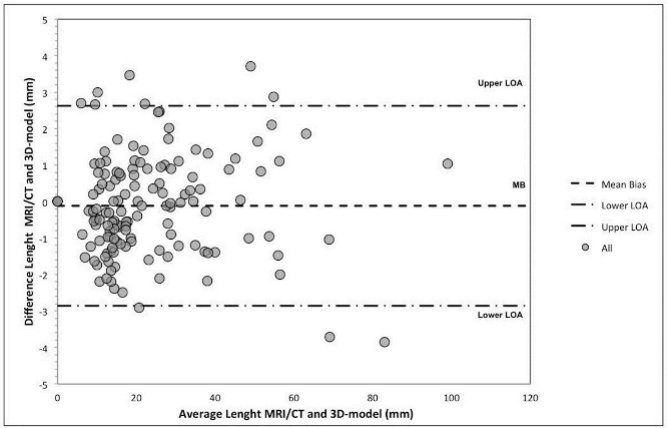
**Bland-Altman analysis of 3D printed model measurement accuracy.** Measurement agreement between 3D printed model direct calliper and medical images (MRI and CT) at analogous anatomical locations. Mean (mean bias of difference), LOA (limits of agreement, ±1.96 standard deviations). Values are expressed as mm.

The Bland-Altman analysis showed accurate agreement in the diameter between medical images and 3D-models (-0.12±1.40 mm, mean bias ± standard deviation, Figure 2). 3D-models showed the spatial relationships between the ventricular septal defect and great vessels (Case2,Case-6,Case-7,Case-8), re-appraisal for biventricular repair (Case-1,Case-8), planning of lateral tunnel completion (Case3), re-opening of a restrictive VSD and its relationship with the conductive tissue (Case4) and evaluation of RVOT aneurysm and pulmonary artery origin (Case 5). Surgeons found the 3D models to be very useful for surgical planning with an overall level of satisfaction of 8.5 out of 10, all agreed (score 4 out of 5) that 3D-models they were helpful to decrease possible surgical complications, strongly agree (score 5 out of 5) that would recommend it to other colleagues as well as teaching for trainees.

## Conclusions

3D-printed cardiovascular models accurately replicate the patient's anatomy and are extremely helpful for planning surgery in complex congenital heart disease. They may potentially reduce operative time and morbi-mortality.

## Funding

This research has been co-financed by Institute of Health Carlos III - FIS research grant number PI13/02319 from the Spanish Ministry of Science and Innovation.

